# Tumor parameters predict the risk of side effects after ruthenium-106 plaque brachytherapy of uveal melanomas

**DOI:** 10.1371/journal.pone.0183833

**Published:** 2017-08-31

**Authors:** Lisa Tarmann, Werner Wackernagel, Domagoj Ivastinovic, Mona Schneider, Peter Winkler, Gerald Langmann

**Affiliations:** 1 Department of Ophthalmology, Medical University Graz, Graz, Austria; 2 Department of Therapeutic Radiology and Oncology, Medical University Graz, Graz, Austria; University of Tasmania, AUSTRALIA

## Abstract

**Background:**

To report on radiation-related side effects and complications after ruthenium-106 plaque brachytherapy of uveal melanomas.

**Methods:**

Medical records of 143 eyes with uveal melanoma, treated by ruthenium-106 brachytherapy between 1997 and 2012 at a single center, were analyzed.

We evaluated the occurrence of radiation-related side effects on the anterior and posterior segment of the eye. The influence of patient, tumor and treatment parameters on outcome was analyzed by multivariate time to event analysis considering competing risks.

**Results:**

The median overall follow-up was 37.9 months.

After treatment, the estimated risk at 12, 24 and 48 months for developing anterior segment complications was 25.3%, 37.5% and 50.3% for cataract formation and 5.4%, 6.4% and 8.1% for secondary glaucoma, respectively.

The estimated risk for the occurrence of posterior segment complications 12, 24 and 48 months after treatment was 3.1%, 6.7% and 18.3% for radiation retinopathy, 18.3%, 27.1% and 42.6% for radiation maculopathy and 16.5%, 21.0% and 32.8% for radiation neuropathy, respectively. The risk of an increase in retinal detachment after treatment was 14.7%, 14.7% and 17.4% at 12, 24 and 48 months, respectively. The risk of vitreous hemorrhage occurring after treatment was 6.2%, 8.1% and 12.7%, and the risk of tumor vasculopathy was 15.4%, 17.4% and 19.0%. Scleral necrosis was observed in one patient.

**Conclusion:**

Radiation-related side effects and complications are common among patients treated with ruthenium brachytherapy for uveal melanoma. However, the risk for those largely depends on individual tumor parameters.

Before treatment, patients should be informed of their specific risks to develop various side effects.

Patient information before treatment should cover not only general information about the treatment and possible complications and side effects but should also give details on the specific risks of the patient in her individual situation. This also includes elucidating the patient's individual resources and expectations and her willingness for long-term regular follow-up examinations and secondary adjunct treatments.

## Introduction

Since eye-conserving radiation therapies have largely replaced enucleation in the treatment of uveal melanoma, the impact of radiation-related side effects, their occurrence and their management have become a main point of interest.

Commonly described side effects include anterior segment complications such as cataract formation, the development of iris neovascularization and secondary glaucoma. Complications affecting the posterior segment of the eye are commonly related to ischemic events of the retina, mainly induced by endothelial cell loss and capillary closure, leading to the formation of teleangiectatic-like channels, capillary collaterals, retinal oedema, microaneurysms and vitreoretinal neovascularizations. [1] These formations are generally summarized as retino-, maculo- and optic neuropathies. Since iodine-125 is the most frequently used radionuclide for plaque brachytherapy literature on the outcome after ruthenium-106 brachytherapy is comparably sparse and only a few reports describing complications after ruthenium-106 plaque brachytherapy, covering selected anterior or posterior segment complications or both, have been published. [2–7]

We analyzed the occurrence of radiation-related side effects and complications of the anterior and posterior segment of the eye after ruthenium-106 plaque brachytherapy for uveal melanoma, comprising cataract formation, secondary glaucoma, retino-, maculo- and optic neuropathies, retinal detachments and/or retinal detachment increases, as well as vitreous hemorrhages.

## Methods

This retrospective analysis comprises the medical records of 143 eyes of 143 patients, out of a total of 172 patients treated with ruthenium (Ru-106) brachytherapy between June 1997 and July 2012 at the Department of Ophthalmology, Medical University of Graz. All data had prospectively been recorded in an electronic database (FileMaker Inc.,v3–6) at our department. This study was approved by the local institutional review board (IRB00002556, Medical University of Graz).

Eye preserving treatment by ruthenium plaque brachytherapy was offered to patients if they gave informed consent and agreed in regularly planned follow up examinations. Further, local tumor characteristics (size, height and location in the eye) had to enable for focal brachytherapy. Relative contraindications comprised large tumor size (longest basal diameter >25 mm, height >7mm) and a close distance of posterior tumor margin to the optic disc or the center of the fovea (within 1.5mm).

Twelve patients had previously been treated by transpupillary thermo-therapy (TTT; n = 11) or gamma knife radiosurgery (GK-RS; n = 1) and were therefore excluded, as were cases with isolated iris melanomas (n = 11) and incomplete medical records (n = 6). Among the included 143 patients, 24 patients (16.8%) had planned combined adjunct TTT (“sandwich-therapy”) 3 months before or after plaque brachytherapy.

The inclusion and exclusion criteria, pre-irradiation assessment, collected descriptive data on tumor, treatment and patient parameters, patient demographics, past medical and family history and the treatment protocol have been described previously. [8] The local tumor control, visual acuity and eye preservation rate of our patients have been published. [8]

### Pre-irradiation assessment

At the first presentation, each patient received a full ophthalmic exam including visual acuity, slit lamp and indirect fundus examination. Moreover, fundus photographs were taken. For accurate determination of the tumor characteristics standardized echography (A/B-scan) was used in all cases. If necessary for diagnosis and/or monitoring of posterior segment complications (e.g. macular edema) during follow-up, ocular angiography (fluorescein and indocyanine green) and optical coherent tomography were performed. Further patient demographics, past medical and family history were evaluated.

### Follow-up

Follow-up examinations were planned approximately one month after the removal of the plaque and thereafter scheduled every three months for the first two years and every six months up to five years after irradiation. Thereafter patients were followed annually. [8] Those follow-up intervals were shortened if necessary. Systemic examinations including blood work, liver parameters and abdominal ultrasound examination were planned every half a year and chest X-ray examinations annually, to rule out metastatic disease.

### Treatment protocol and plaque surgery

#### Irradiation protocol

uveal intraocular melanomas (excluding isolated iris melanomas) were treated using local ruthenium-106 plaque brachytherapy. According to the wide spectrum of melanomas in study population (longest basal diameter and tumor height) we used different plaque types, depending on the basal diameter. The following plaques were used: CCA (up to 15.3mm), CCB (up to 20.2mm), CCC (up to 24.8mm), CIB (up to 20.2mm), CCX (up to 11.6mm), COB (up to 19.8mm), manufactured by BEBIG, Berlin, Germany. The treatment was planned to deliver a minimum irradiation dose to the tumor apex of at least 100 Gy but not to exceed a maximum dose to the outer scleral surface of 1000 Gy.

#### Surgical intervention

The plaque insertion and positioning surgery was usually done in general anesthesia, the plaque removal usually in local retrobulbar anesthesia. To achieve a proper position of the radioactive plaque on the globe a non-radioactive transparent plastic template of the same size (dummy applicator) was used. Indirect ophthalmoscopy with scleral indentation and transpupillary transillumination were used to visualize the tumor and outline the tumor borders on the scleral surface with a surgical pen. Extraocular muscles (rectus and oblique muscles) were removed and reinserted if necessary. The applicator was positioned on the sclera with minimal safety margin of one millimeter around the tumor margins.

### Outcome measures

The primary outcome measures were complications of the anterior and posterior segment of the eye.

All complications were analyzed considering pre-irradiation tumor parameters and treatment parameters. A Kaplan-Meier analysis was used to estimate the event rate in case of unequal follow up times. Competing risk regression (Fine-Gray method) was done to include competing and censoring events into the risk calculation. The end of follow-up was defined as the day of the last presentation of the patient at our department or as the day of enucleation of the affected eye regardless of causality.

#### Definitions of complications

Cataract formation due to radiotherapy was defined as any asymmetrical increase in lens opacity on the treated eye. Secondary glaucoma was defined as any unilateral increase in the previously normal intraocular pressure (IOP) on the treated eye if the IOP of the other eye stayed within normal ranges. Radiation retinopathy was defined as any occurrence of occlusions and changes of the retinal vessels, such as microinfarcts, hemorrhages, microaneurysms, retinal edema and lipid exudations. A radiation maculopathy was defined as showing the same features within a 1.5 disc diameter of the foveola. Optic neuropathy was defined as partial or complete atrophy of the optic nerve head with or without bleeding at the optic disc. Isolated tumor vasculopathy was defined as bleeding or exudation on the tumor surface.

### Statistical analysis

Statistical analysis was performed using Stata 13.0 (Stata Inc., College Station, TX). The values of continuous variables are presented as the median, range and interquartile range (IQR) or standard deviation (SD). Categorical variables are presented as relative and absolute frequencies. Follow-up was calculated by the inverse Kaplan-Meier method. [9] The occurrence of radiation-related complications after treatment was calculated using the Kaplan-Meier method. For each complication, censoring events, which made further clinical evaluation of the complication event impossible, were defined and considered in the analysis (see Table 1)).

**Table 1:** Tabulation of censoring events for each complication.

Complication	Censoring event
*Cataract*	Vitrectomy without endoresectionVitrectomy with endoresectionSecondary brachytherapyGamma knife radiosurgery
*Glaucoma*	Vitrectomy without endoresectionVitrectomy with endoresectionSecondary brachytherapyGamma knife radiosurgery Transscleral cyclocryotherapyTransscleral cyclophotocoagulationRetinal laser photocoagulationIntravitreal anti-VEGF injection
*Retinopathy*	Vitrectomy without endoresectionVitrectomy with endoresectionSecondary brachytherapyGamma knife radiosurgery Retinal laser photocoagulationIntravitreal anti-VEGF injection
*Maculopathy*	Vitrectomy without endoresectionVitrectomy with endoresectionSecondary brachytherapyGamma knife radiosurgery Retinal laser photocoagulationIntravitreal anti-VEGF injection
*Optic neuropathy*	Secondary brachytherapyGamma knife radiosurgery
*Retinal detachment*	Vitrectomy without endoresectionVitrectomy with endoresectionSecondary brachytherapyGamma knife radiosurgery
*Tumor vasculopathy*	Vitrectomy without endoresectionVitrectomy with endoresectionSecondary brachytherapyGamma knife radiosurgery
*Vitreous hemorrhage*	Vitrectomy without endoresectionVitrectomy with endoresectionSecondary brachytherapyGamma knife radiosurgery Biopsy

Table 1: showing the censoring events for each complication included in the analysis.

Competing risk regression was used to calculate the cumulative incidence function (CIF) for each complication and to perform a uni- and multivariate risk factor analysis as described previously. [8] All statistically significant variables found in the univariate risk analysis were included into the multivariate risk calculation. P values of ≤ 0.05 were defined as statistically significant.

## Results

### Patient- and tumor- parameters

#### Demographic data

From 143 records reviewed, 81 (56.6%) patients were female and 62 (43.4%) patients were male gender. The age at the first presentation was 62.5 years (median; IQR: 53.4–74.0; range: 28.6–88.7) (see Table 2).

**Table 2:** Patient demographics.

*Characteristic*	Value (%)
Sex	
Male	62 (43.4)
Female	81 (56.6)
Laterality	
Right	71 (49.6)
Left	72 (50.3)
Age (years)	62.50 (median; IQR: 53.4–74.0; range: 28.6–88.7)
Follow up (months)	37.88 (median; IQR: 20.37–62.0)
***Systemic diseases***	
Systemic diabetes	16 (11.2)
Systemic hypertension	60 (42.0)
Coronary artery disease	14 (9.8)
Other systemic cancer	13 (9.1)
***Ocular abnormalities of the affected eye***	
Retinal detachment	68 (47.5)
Cataract	59 (41.2)
Pseudophakia or aphakia	13 (9.1)
Glaucoma	4 (2.8)
Choroidal nevus	3 (2.1)

Table 2: Overview about patient characteristics: IQR = interquartile range.

#### Past medical history

11.2% (n = 16) of patients suffered from systemic diabetes, 42% (n = 60) from systemic hypertension, 9.8% (n = 14) from a coronary artery disease and 9.1% (n = 13) from another systemic cancer.

#### Ocular status

The right eye was affected in 49.7% (n = 71) of the patients, the left eye in 50.3% (n = 72) of the patients.

A concurrent retinal detachment was diagnosed in 47.5% (n = 68) of the patients, an age related macular degeneration in 4.9% (n = 7) and a concurrent diabetic retinopathy in one patient (0.7%) at time of diagnosis.

41.2% (n = 59) of patients had a pre-existing cataract and 9.1% (n = 13) of patients were pseudophacic at the initial presentation. A further 2.8% (n = 4) showed to have pre-existing glaucoma. Three patients (2.1%) had another choroidal nevus in the affected eye.

#### Initial visual acuity

The initial visual acuity before treatment was at least 20/40 in 73.4% (n = 105) of cases, in 96.5% (n = 138) at least 20/200 and in 98.6% (n = 141) at least counting fingers (CF).

#### Tumor parameters

The median tumor height was 4.5 mm (IQR: 3.8–6.6 mm) and the longest basal diameter of the lesions examined was 11.0 mm (median; IQR: 9.5–13.4). The median distance of the tumor to the optic disc was 6 mm (IQR: 3.7–9.9) and to the center of the macula region 7.5 mm (median; IQR: 3.7–10.5). All patients were categorized into TNM7 groups [10]. 36 (25.2%) tumors were classified in T1, 70 (48.9%) tumors in T2, 32 (22.4%) in T3 and 5 (3.5%) in T4 category. 118 (82.5%) lesions were dome- (114 cases) or plateau- (4 cases) shaped and 25 (17.5%) tumors showed a mushroom phenotype. 92.3% (132) of the lesions were melanotic and 7.7% (11) of lesions were amelanotic.

The anterior tumor margin of the most melanomas examined was located anterior to the equator (n = 79, 55.2%); In 36 (25.2%) cases posterior to the equator and in 28 (19.6%) anteriorly at the ciliary body. The posterior margin of the lesions evaluated was within 1.5 mm from the optic disc or the center of the macula in 11.2% (n = 16), in 21.7% (n = 31) between 1.6 and 3.0 mm from the optic disc or fovea, in 58.7% (n = 84) posterior to equator (≥ 3mm to optic disc or fovea) and in 8.4% (n = 12) anterior to equator of the eye. [8]

#### Treatment parameters

The median delivered dose to the external scleral surface was 689.8 Gy (IQR: 488.7–922.8) and the median delivered dose to the tumor apex was 89.8 Gy (IQR: 59.3–120.8). The applied dose to the tumor apex was < 80 Gy in 60 (42%) cases, 80–110 Gy in 37 (26%) cases and > 110 Gy in 46 (32%) cases. The dose to the scleral surface was < 200Gy in 1 (0.7%) case, 200–500 Gy in 37 (26%) cases, 500–800 Gy in 47 (33%) cases and >800 Gy in 58 (41%) cases. Twenty-four patients received an adjunct sandwich therapy, which was planned and performed in case of uncertain irradiation sufficiency, mainly in case of extended tumor height. The first application was performed in April 1997. [8]

#### Post-irradiation adjunct treatments

An overview about the performed post irradiation adjunct treatments is summarized in Table 3.

**Table 3:** Summary of post-irradiation adjunct treatments.

Characteristic	Group A Value (%)n = 99	Group B Value (%)n = 44
Cyclocryotherapy	0	1 (2.27)
Enucleation	3 (3.03)	7 (15.91)
Photodynamic therapy	1 (1.01)	0
Ru-106 brachytherapy	2 (2.02)	4 (9.09)
Retinal cryotherapy	0	2 (4.55)
Retinal photocoagulation	7 (7.07)	7 (15.91)
TSCPC	1 (1.01)	0
TTT for exsudation	4 (4.44)	3 (6.82)
TTT sandwich	5 (5.05)	19 (43.18)
TTT for recurrence	5 (5.05)	2 (4.55)
TTT for other reason	1 (1.01)	2 (4.55)
Vitrectomy with endoresection	1 (1.01)	8 (8.18)
Vitrectomy without endoresection	6 (6.06)	5 (11.36)
Anti-VEGF intravitreal treatment	13 (13.13)	4 (9.09)

Table 3. showing an overview of adjunct treatments performed after ruthenium-106 plaque brachytherapy. TTT = transpupillary thermotherapy; "TTT for other reason" was performed in case of insufficient scaring along the tumor margin; TSCPC = transscleral cyclophotocoagulation; VEGF = vascular endothelial growth factor.

#### Follow up time

The median overall follow-up time, calculated by the inverese Kaplan-Meier method, was 37.88 months (median; IQR: 20.37–62.0).

### Complications of the anterior and posterior segment of the eye

The majority of patients 76.9% (n = 110) developed at least one side effect or complication after treatment (see Table 4). The mean number of complications was 1.9 (SD: 1.7).

**Table 4:** Radiation related complications.

Complication	Overall value (%)	1 year post irradiation (CI 95%)	2 years post irradiation (CI 95%)	4 years post irradiation (CI 95%)
Cataract	58 (40.6%)	25.3% (18.5–34.0%)	37.5% (29.2–47.2%)	50.3% (39.6–62.1)
Glaucoma	15 (10.5%)	5.4% (2.6–10.9%)	6.4% (3.2–12.5%)	8.1% (4.1–15.6%)
Retinopathy	24 (16.8%)	3.1% (1.2–8.1%)	6.9% (3.5–13.4%)	18.3% (11.0–29.5%)
Maculopathy	55 (38.5%)	18.3% (12.5–26.2%)	27.1% (19.9–36.2%)	42.6% (32.9–53.8%)
Optic neuropathy	36 (25.2%)	16.5%(11.1–23.9%)	21.0% (14.9–29.2%)	32.8% (24.4–43.1%)
Retinal detachment	27 (18.9%)	14.7% (9.7–21.8%)	14.7% (9.7–21.8%)	17.4% (11.6–25.5%)
Vitreous hemorrhage	25 (17.5%)	6.2% (3.2–12.1%)	8.1% (4.4–14.6%)	12.7% (7.3–21.6%)
Tumor vasculopathy	30 (21.0%)	15.4% (10.2–22.9%)	17.4% (11.8–25.4%)	19.0% (12.9–27.5)

Table 4: showing an overview of radiation-induced anterior and posterior segment complications (number of cases and values estimated by Kaplan-Meier method).

#### Cataract formation

Overall, we observed 58 (40.6%) patients who developed a secondary cataract after treatment. The estimated risk of cataract formation 12, 24 and 48 months after irradiation was 25.3% (CI 95%: 18.5–34.0%), 37.5% (CI 95%: 29.2–47.2%), and 50.3% (CI 95%: 39.6–62.1), respectively.

Risk factors found in the uni- and multivariate analysis are shown in Table 5. The only statistically significant factor, which remained in the multivariate analysis for cataract development was an anterior tumor location (posterior margin anterior to the equator of the eye).

**Table 5:** Risk factors for cataract formation. Univariate and multivariate risk factor analysis.

		95% CI for hazard ratio		
	HR	Lower	Upper	p value
Univariate analysis				
Treatment dose ≥90 Gy				0.0137*
Posterior tumor margin				
1–2 DD from disc or foveola	1.6	0.6	4.3	0.339
posterior to the equator	1.4	0.6	3.4	0.408
anterior to the equator	3.3	1.3	8.3	0.013*
Dtd	1.0	1.0	1.1	0.055
Dtf	1.04	1.0	1.1	0.087
Pre-existing cataract	0.6	0.3	1.0	0.071
Pre-existing cataract f.e.	0.5	0.3	1.0	0.045*
Multivariate analysis				
Posterior tumor margin				
1–2 DD from disc or foveola	1.6	0.6	4.3	0.339
posterior to the equator	1.4	0.6	3.4	0.408
anterior to the equator	3.3	1.3	8.3	0.013*

Table 5: showing the results of the univariate and multivariate risk factor analysis for cataract formation. Treatment dose ≥90 Gy = total treatment dose to the tumor apex ≥ 90 Gray; f.e. = fellow eye; Dtd = tumor distance to disc; Dtf = tumor distance to foveola; CI = confidence interval; HR = hazard ratio; Gy = Gray; DD = disc diameter; 1–2 DD to disc or foveola = 1–2 disc diameter distance of the posterior tumor margin to the optic disc or the center of the macula

* values marked by "*" showed to be statistically significant.

#### Secondary glaucoma

After treatment, in total 15 (10.5%) patients developed a secondary glaucoma. The estimated risk of an increase of IOP was 5.4% (CI 95%: 2.6–10.9%), 6.4% (CI 95%: 3.2–12.5%), and 8.1% (CI 95%: 4.1–15.6%) at 12, 24, and 48 months after irradiation. Uni- and multivariate risk factors are shown in Table 6. Clinically detectable neovascularisation of the iris was found in 2.2% (CI 95%: 0.7–6.6%), 3.1% (CI 95%: 1.2–8.3%), and 4.8% (CI 95%: 1.9–11.7%) of cases at 12, 24, and 48 months after treatment. No statistically significant risk factors were found.

**Table 6:** Risk factors for intraocular pressure increase. Univariate and multivariate risk factor analysis.

		95% CI for hazard ratio		
	HR	Lower	Upper	P value
Univariate analysis				
Tumor shape	5.8	1.6	21.2	0.007
Sandwich TTT	5.1	1.6	21.6	0.006
Dtd	1.1	1.0	1.3	0.023
Multivariate analysis				
Sandwich TTT	16.0	3.4	74.3	<0.0001
Dtd	1.2	1.1	1.4	0.002

Table 6: showing the results of univariate and multivariate risk factor analysis for a secondary increase in intraocular pressure. Dtd = tumor distance to disc; tumor shape = mushroom/dome; sandwich TTT = combined transpupillary thermotherapy; CI = confidence interval; HR = hazard ratio

#### Radiation-induced retinopathy, maculopathy and optic neuropathy

After treatment, we observed 24 (16.8%) patients to develop a radiation retinopathy, 55 (38.5%) patients to develop a radiation maculopathy and 36 (25.2%) patients to develop a radiation related optic neuropathy, respectively. The estimated risk 12, 24 and 48 months after irradiation of the development of radiation retinopathy was 3.1% (CI 95%: 1.2–8.1%), 6.9% (CI 95%: 3.5–13.4%) and 18.3% (CI 95%: 11.0–29.5%), respectively. The risk of radiation maculopathy was 18.3% (CI 95%: 12.5–26.2%), 27.1% (CI 95%: 19.9–36.2%), and 42.6% (CI 95%: 32.9–53.8%), and the risk of a radiation optic neuropathy was 16.5% (CI 95%: 11.1–23.9%), 21.0% (CI 95%: 14.9–29.2%) and 32.8% (CI 95%: 24.4–43.1%), respectively.

The risk factors found in the univariate and multivariate analysis are shown in Table 7.

**Table 7:** Risk factors for the development of radiation retino-, maculo- and optic neuropathy. Univariate and multivariate risk factor analysis.

		95% CI for hazard ratio		
	HR	Lower	Upper	p value
**Radiation retinopathy**				
Univariate analysis				
Treatment dose ≥ 90 Gy	2.9	1.0	8.2	0.046
Tumor height	0.8	0.6	1.0	0.047
Multivariate analysis				
Treatment dose ≥ 90 Gy	2.9	1.0	8.2	0.046
**Radiation maculopathy**				
**Radiation maculopathy**				
Univariate analysis				
Tumor shape	2.7	1.4	5.3	0.004
Tumor height	1.1	1.0	1.2	0.037
Tumor LBD	1.1	1.0	1.2	0.049
Dtd	0.9	0.8	1.0	0.005
Dtf	0.9	0.9	1.0	0.002
Pre-existing RD	3.3	1.8	6.0	<0.001
Initial VA	0.9	0.8	1.0	0.056
Multivariate analysis				
Pre-existing RD	3.2	1.7	6.0	<0.001
Tumor shape	2.5	1.4	4.8	0.004
**Optic neuropathy**				
Univariate analysis				
Treatment dose ≥ 90 Gy				0.035
Dose to tumor apex	0.98	0.98	0.99	0.005
Dose to disc	1.002	1.001	1.002	<0.001
TNM 3–4	2.9	1.5	5.7	0.001
Treatment dose ≥ 90 Gy	0.5	0.2	1.0	0.049
Anterior tumor location	0.3	0.1	1.0	0.031
Dtd <1.5 mm	0.1	0.04	0.3	<0.001
Dtf	0.9	0.9	1.0	0.041
Tumor height	1.2	1.1	1.3	<0.001
Tumor shape	1.9	0.9	3.8	0.077
Tumor LBD	1.2	1.1	1.3	0.001
Pre-existing RD	2.5	1.3	4.9	0.006
Sandwich TTT	2.1	0.9	4.6	0.064
Multivariate analysis				
Dtd <1.5 mm	0.1	0.04	0.2	<0.001
Tumor LBD	1.2	1.1	1.3	<0.001

Table 7: showing the risk factors for the development of radiation retino- maculo- and optic neuropathy found in the univariate and multivariate risk factor analysis. Anterior tumor location = posterior tumor margin is located anterior to the equator; treatment dose ≥ 90 Gy = total treatment dose to the tumor apex ≥ 90 Gray; Dtd = tumor distance to disc; Dtf = tumor distance to foveola; tumor LBD = tumor longest basal diameter; Gy = Gray; pre-existing RD = pre-existing retinal detachment before treatment; tumor shape = mushroom/dome; sandwich TTT = combined transpupillary thermotherapy; TNM 3–4 = tumor, node, metastasis classification version 7, TNM3/4 vs. TNM1/2 (Kivela T, Kujala E. Prognostication in eye cancer: the latest tumor, node, metastasis classification and beyond. Eye 2013;27(2):243–52); initial VA = initial visual acuity before treatment; CI = confidence interval; HR = hazard ratio

#### Post-treatment retinal detachment and vitreous hemorrhage

In total we observed 27 (18.9%) patients, who developed a retinal detachment, or retinal detachment increase and 25 (17.5%) patients, who developed a vitreous hemorrhage after treatment. The estimated risk 12, 24 and 48 months after irradiation of the development of a retinal detachment or an increase in retinal detachment was 14.7% (CI 95%: 9.7–21.8%), 14.7% (CI 95%: 9.7–21.8%), and 17.4% (CI 95%: 11.6–25.5%) and for a vitreous hemorrhage was 6.2% (CI 95%: 3.2–12.1%), 8.1% (CI 95%: 4.4–14.6%), and 12.7% (CI 95%: 7.3–21.6%).

The risk factors found in the univariate and multivariate risk factor analysis are shown in Table 8.

**Table 8:** Risk factors for the development of a postoperative retinal detachment (RD) or RD increase compared to pre-irradiation values and vitreous hemorrhage occurrence. Univariate and multivariate risk factor analysis.

			95% CI for hazard ratio			
	HR		Lower		Upper	P value
**Retinal detachment**						
Univariate analysis						
Tumor size	2.3		1.0		5.4	0.055
Tumor height	1.2		1.1		1.4	0.002
Tumor LBD	1.1		1.0		1.3	0.067
Dtd <1.5 mm	0.2		0.1		0.6	0.007
Dtf	0.9		0.8		1.0	0.026
Tumor shape	3		1.3		7.3	0.012
Pre-existing RD	4.2		1.6		10.9	0.003
Treatment dose rate (Gy/h)	1.2		1.0		1.5	0.016
Initial VA	0.9		0.8		1.0	0.010
Multivariate analysis						
Tumor height	1.2		1.0		1.3	0.052
Pre-existing RD	4.0		1.3		11.9	0.013
Treatment dose rate (Gy/h)	1.3		1.1		1.6	0.007
Dtd <1.5 mm	0.3		0.1		0.9	0.040
**Vitreous hemorrhage**						
Univariate analysis						
Tumor shape	5.4		1.8		15.7	0.002
Tumor height	1.2		1.1		1.4	0.006
Sandwich TTT	3.8		1.3		11.2	0.015
Dose to apex	0.98		0.97		1.0	0.053
Multivariate analysis						
Tumor shape	5.4		1.8		15.7	0.002

Table 8: showing the risk factors for the development of RD or RD increase compared to pre-irradiation values and the occurrence of vitreous hemorrhage after irradiation, found in the univariate and multivariate risk factor analysis. Dtd = tumor distance to disc; Dtf = tumor distance to foveola; tumor LBD = tumor longest basal diameter; Gy = Gray; h = hour; pre-existing RD = RD existing before treatment; tumor shape = mushroom/dome; sandwich TTT = combined transpupillary thermotherapy; initial VA = initial visual acuity before treatment

#### Tumor vasculopathy

During follow-up, we observed 30 (21.0%) patients showing a tumor vasculopathy. The estimated risk of the development of postoperative tumor vasculopathy at 12, 24 and 48 months after irradiation was 15.4% (CI 95%: 10.2–22.9%), 17.4% (CI 95%: 11.8–25.4%) and 19.0% (CI 95%: 12.9–27.5), respectively. No statistically significant risk factors could be found.

## Discussion

In this study, we describe anterior and posterior segment complications after ruthenium-106 brachytherapy.

Due to the small number of studies evaluating the side effects after ruthenium-106 plaque brachytherapy and the differences in the inclusion criteria and definition of the complications, a valid comparison between these studies and our study is difficult.

For the evaluation and calculation of the side effects, several factors had to be considered. First, we corrected for unequal follow-up times. Second, the occurrence of one complication or its treatment can hinder the identification of another. For that reason, such situations were considered “censoring events” and taken into account in our analysis. At the time point of the necessary subsequent surgical intervention, the analysis of the respective complications was stopped and the patient considered “censored”. Finally, secondary enucleation after treatment also defines the end of follow-up and was regarded as a “competing risk”.

Cataract formation: The most frequent complication, occurring in 40.6% (n = 58) of our study population with an estimated risk of 50.3% after 4 years is secondary cataract formation. Other authors also identified cataract formation as one of the most frequent complications after ruthenium-106 brachytherapy, occurring in up to 37% [3] and 33% [11] of cases, as well as 21% and 28% three and five years after treatment [5]. Summanen et al. reported the estimated risk of secondary cataract formation to be 21%, 27% and 37% at 2, 3 and 5 years after treatment. In that study, the most important risk factors were tumor size and anterior tumor location. In our study, no correlation to the tumor size could be found. The most important risk factors were anterior tumor location, increasing the risk of cataract formation by two-fold, and an elevated irradiation dose (≥ 90 Gy) to the tumor apex.

The higher rate of irradiation-induced cataracts in our series might be explained by the difficulty of differentiation between age-related cataract formation and irradiation-induced cataract formation due to the retrospective study design.

Secondary glaucoma: The estimated risk of the occurrence of secondary glaucoma 12, 24 and 48 months after irradiation was 5.4%, 6.4%, and 8.1%, respectively. The most important risk factors for post-irradiation IOP increase were a mushroom-like tumor shape and a higher tumor distance to the disc (p = 0.032), with a 4-fold increase in risk if the tumor was ≥ 10 mm away from the optic disc.

Other authors found similar results for IOP increase after irradiation, at 7% [4] or 3.9% within the first 5 years after treatment [3]. Summanen et al. found the estimated risk of the development of secondary glaucoma to be 4%, 9% and 19% at 2, 3 and 5 years after treatment. [2] In contrast to this study, tumor category (TNM) was not identified as risk factor in our series.

Radiation retinopathy: In our population, the estimated risk of the development of a radiation-induced retinopathy was 18.3% as of four years after treatment.

We could not find any clinically relevant risk factors for the development of a radiation retinopathy. The main risk factor was a reduced tumor size and, correspondingly, an elevated irradiation dose to the tumor apex. This surprising result might be explained by the higher number of adjunct treatments in the case of larger tumors, which might have influenced the results of the statistical analysis (as those cases were considered to be censored) but also might have affected the occurrence of radiation retinopathy, as the result remained unchanged even if those cases were retained in the analyses (e.g., vitrectomy and extensive laser coagulation might prevent the appearance of retinopathies [12, 13]). Interestingly, Naseripour et al. also found, in addition to the tumor location (choroidal versus ciliochoroidal) that a reduced tumor height (HR: 0.55) was a statistically significant risk factor for the development of a radiation retinopathy, which occurred in 39.2% (non-proliferative radiation retinopathy) and in 13.7% (proliferative radiation retinopathy) ten years after the treatment. [3] Consistently, Browne et al. observed the occurrence of a radiation-induced retinopathy in 20% of treated cases. [11]

Radiation maculopathy: In our retrospective data analysis, we evaluated radiation-induced maculopathies separately. The estimated occurrence 1, 2 and 4 years after treatment was 18.3%, 27.1% and 42.6%, respectively.

Other authors described similar rates. Summanen et al. found this complication in 15% of cases at two years and 30% at three years after treatment [2]. Others report the incidence of radiation maculopathies to be 19.6% at 10 years after treatment [3], 29% after a median time interval of 13.5 months [4] or 14% at three and 28% at five years after irradiation [5].

The risk factors found in our evaluation comprised a mushroom-like tumor shape, a reduced tumor distance to the disc or fovea, and pre-existing retinal detachment (Table 4). Summanen et al. also reported a significantly reduced risk with a greater tumor distance to the fovea. [2] In the multivariate analysis, mushroom tumor shape and a pre-existing retinal detachment remained statistically significant.

Optic neuropathy: We found radiation induced optic neuropathies in 16.5%, 21.0% and 32.8% 12, 24, and 48 months after irradiation. The rate of optic neuropathies reported in the literature varies widely.

Accordingly, Summanen et al. reported incidence rates of 10% and 12% at two and three years after treatment [2], whereas Naseripour et al. describe rates of up to 32% at five and 10 years after irradiation [3]. Another study with 93 evaluated cases with juxtapapillary melanomas revealed relatively high incidence rates of complete radiation optic neuropathies in 23% and 53% and partial optic neuropathies in 66% and 82% of cases five and ten years after ruthenium-106 plaque brachytherapy. [6] The most important risk factors seem to be the tumor location close to the optic disc and an elevated irradiation dose to the disc. [2, 3, 6]

Similarly, in our analysis, the most important risk factors were a small tumor distance to the disc (<1.5 mm) and, in association, an elevated longest basal diameter of the tumor.

Vitreous hemorrhage and retinal detachment: The risk of developing a vitreous hemorrhage was 12.7%, and the risk of a retinal detachment (RD) or an increase in a pre-existing RD was 17.4% as of four years after irradiation. Other authors describe similar rates, with RD developing or increasing in 26% of cases by five years after treatment, primarily in large tumors. [2]

We found pre-existing retinal detachments (4-fold risk elevation) and the treatment dose rate to be the most important statistically significant risk factors in the multivariate analysis.

Limitations of our study: As with all retrospective studies we cannot prove a causal association between the identified risk factors and the respective complication. In addition, our study covers a long time span. Though data have been recorded in a prospective manner in a clinical database with defined criteria, the possibility remains that some of these have been applied differently over time. Using the available photographs, ultrasounds and angiographic images, we checked for correctness to keep this risk as small as possible. Finally, due to the relatively small number of cases some risk factors might not have been detected.

Comparison of iodine-125 and ruthenium-106 plaque brachytherapy Iodine (I-125) is the most commonly used radionuclide for the brachytherapy of uveal melanomas. We assume that the treatment of uveal melanomas by Ru-106 plaques is less frequent because this nuclide is limited in penetration depth and therefore commonly considered suitable for uveal melanomas up to a maximal height of 6 mm. However, it has also been used for significantly higher tumors by some authors. [14] A further reason for the wider distribution and worldwide use of iodine (I-125) plaques may be the use of these applicators in the Collaborative Ocular Melanoma Study (COMS), which produced a large amount of treatment evidence. Iodine-125 emits low-energy gamma radiation, resulting in a higher penetration depth than ruthenium-106 but also a higher radiation dose to surrounding healthy tissues. [15] Wilkinson et al. performed a dosimetric comparison and showed significantly less mean radiation doses to the macula, optic disk and lens (18%, 53% and 89%) for ruthenium. [15] These differences in case selection and in the dose to the surrounding healthy tissue might explain the differences in reported complication rates but also render a direct clinical comparison of these two radionuclides extremely difficult.

## Conclusion

Anterior and posterior segment complications are common after the irradiation of choroidal and ciliary body melanomas by ruthenium brachytherapy. The most important risk factors for radiation-induced side effects seem to be the tumor size and localization, influencing the irradiation dose to important vulnerable structures.

As complications might occur years after initial treatment, long-term follow-up must be ensured.

Before treatment, patients should be informed of their specific risks to develop various side effects.

Patient information before treatment should cover not only general information about the treatment and possible complications and side effects but should also give details on the specific risks of the patient in her individual situation. This also includes elucidating the patients individual resources and expectations and her willingness for long term regular follow-up examinations and secondary adjunct treatments.

## Supporting information

S1 File Minimal anonymized data set. (TXT)
